# Acute angle-closure glaucoma in retinopathy of prematurity following pupil dilation

**DOI:** 10.1186/s12886-015-0099-7

**Published:** 2015-08-08

**Authors:** Shiu-Chen Wu, Yung-Sung Lee, Wei-Chi Wu, Shirley H L Chang

**Affiliations:** Department of Ophthalmology, Chang Gung Memorial Hospital, 5 Fu-Hsin Rd., Kweishan 333, Taoyuan, Linkou Taiwan; Chang Gung University, College of Medicine, Taoyuan, Taiwan

## Abstract

**Background:**

Pupil dilation is a known risk factor for acute angle-closure glaucoma. Regular retinal evaluation is necessary for retinopathy of prematurity (ROP) cases. An acute attack of angle-closure glaucoma following pupil dilation in regressed ROP has never been reported.

**Case presentation:**

A five-year-old girl presented to the hospital for a routine retina check-up. The patient was born prematurely with a gestation age of 27 weeks and a body weight of 980 grams. She had a history of stage 4A ROP in the right eye and received scleral buckling. After pupil dilation with 1 % tropicamide and 10 % phenylephrine for retinal examination, acute elevation of intraocular pressure (IOP) was observed in the right eye. Her IOP remained over 50 mmHg in the right eye even under treatment with oral acetazolamide and maximal tolerated doses of topical anti-glaucoma medications. Ultrasound biomicroscopy (UBM) showed that the angle in the right eye was closed 360 degrees circumferentially. In order to lower IOP, trabeculectomy with mitomycin C (0.2 mg/cc) was performed under general anesthesia. Postoperatively, the cornea became clear, the filtering bleb functioned well, and IOP returned to normal values. In the two-year follow-up, IOP was kept around 15 mmHg without anti-glaucoma medications. Although mild lens opacity was noted, her postoperative VA remained 20/200 in the right eye.

**Conclusion:**

Regular retinal evaluation will be necessary for the increasing number of ROP cases to be seen in the future. Ophthalmologists should bear in mind that pupil dilation for a retina check-up could result in acute angle-closure glaucoma in ROP patients.

## Background

Angle-closure glaucoma has been previously reported to be associated with retinopathy of prematurity (ROP). We report a case of regressed ROP who experienced an acute attack of angle-closure glaucoma in the right eye after pupil dilation for a routine retinal examination. To the best of our knowledge, an acute attack of angle-closure glaucoma following pupil dilation in regressed ROP has never been reported. Regular retinal evaluation will be necessary for the increasing number of ROP cases to be seen in the future. In such cases, the pupil should be dilated with caution.

## Case presentation

**Fig. 1:**
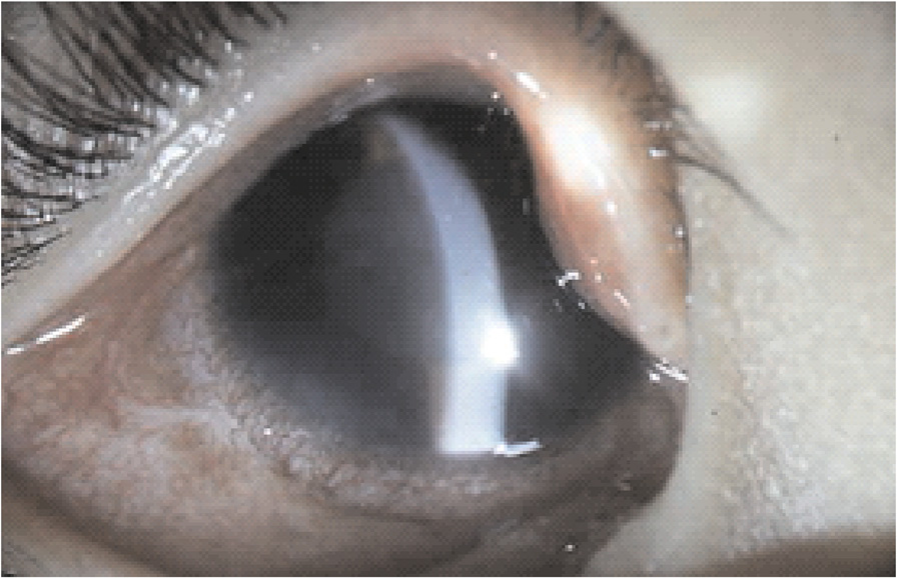
An acute attack of angle-closure glaucoma following pupil dilation for retinal examination in a five-year-old girl with regressed retinopathy of prematurity. Typical presentations of acute angle-closure glaucoma, including edematous cornea, shallow anterior chamber, fixed-dilated pupil, and glaucomatous flecks of the lens, were observed

**Fig. 2:**
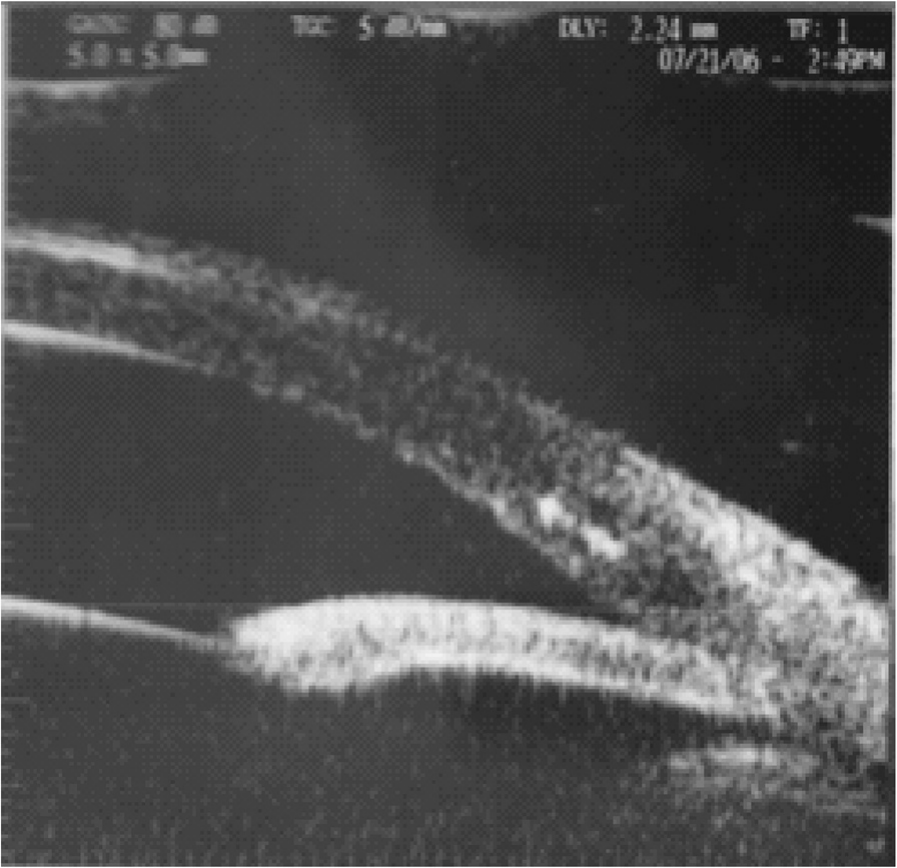
Ultrasound biomicroscopy (UBM) findings related to an acute angle-closure attack in a five-year-old girl. UBM showed the angle in the right eye was closed at 360 degrees circumferentially

**Fig. 3:**
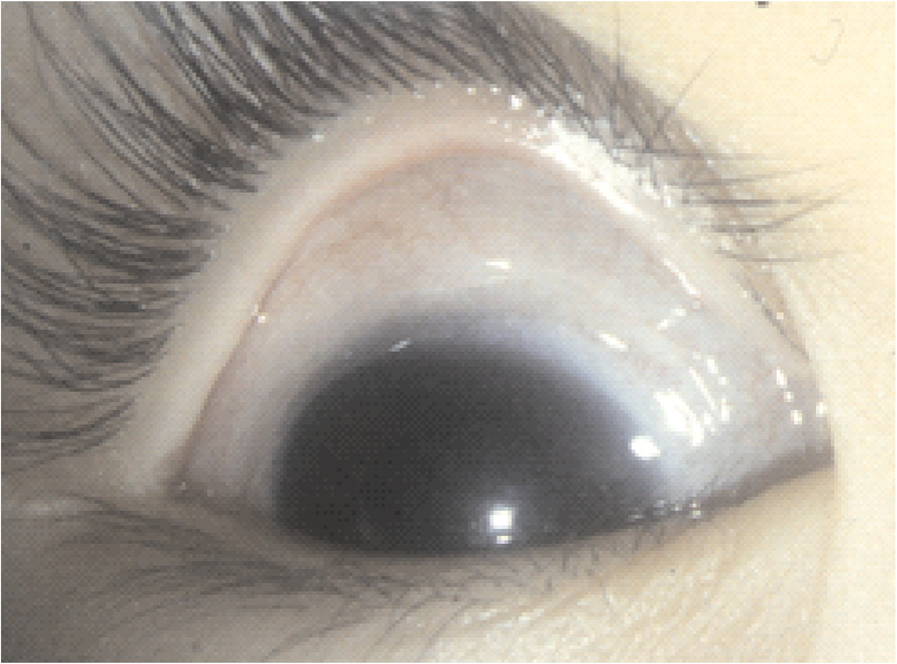
Postoperative view of the external eye. The cornea became clear, the filtering bleb functioned well, and IOP returned to normal values

A five-year-old girl presented to the Department of Ophthalmology, Chang-Gung Memorial Hospital, Taoyuan, Taiwan for a routine retina check-up. The patient was born prematurely with a gestation age of 27 weeks and a body weight of 980 grams. She received laser treatment for stage 3 ROP. Despite laser treatment, her retinopathy progressed to stage 4A ROP in both eyes. She received scleral buckling for both eyes. The retina was nicely attached in the right eye after scleral buckling, but the retina remained detached and progressed to stage 5 ROP in the left eye. Her prior visual acuity (VA) was 20/200 in the right eye; the left eye displayed no light perception with phthisical changes. After pupil dilation with 1 % tropicamide and 10 % phenylephrine for retinal examination, acute elevation of intraocular pressure (IOP) was observed in the right eye. Typical presentations of acute angle-closure glaucoma, including edematous cornea, shallow anterior chamber, fixed-dilated pupil, and glaucomatous flecks of the lens, were observed (Fig. 1). IOP rose to 50 mmHg. Visual acuity in the right eye dropped to finger counting. Her IOP remained over 50 mmHg in the right eye even under treatment with oral acetazolamide and maximal tolerated doses of topical anti-glaucoma medications. Ultrasound biomicroscopy (UBM) (P40; Paradigm Medical Industries, Inc. Salt Lake City, UT) showed that the angle in the right eye was closed 360 degrees circumferentially (Fig. 2). In order to lower IOP, trabeculectomy with mitomycin C (0.2 mg/cc) was performed under general anesthesia. Postoperatively, the cornea became clear, the filtering bleb functioned well, and IOP returned to normal values (Fig. 3). In the two-year follow-up, IOP was kept around 15 mmHg without anti-glaucoma medications. Although mild lens opacity was noted, her postoperative VA remained 20/200 in the right eye.

## Discussion

Factors contributing to the development of angle-closure glaucoma in ROP have been proposed [1–5]. Persistent fetal vasculature and retrolental fibrotic membranes may push the lens-iris diaphragm forward and compromise the angle structure. In patients who undergo the scleral buckling procedure, the buckle can displace the lens and iris forward, which may cause the anterior chamber to become even more shallow. Pupil dilation could aggravate the anterior chamber shallowness and is a known risk factor for acute angle-closure glaucoma [6]. Such pre-existing conditions may have affected the development of the current case, including the acute pupillary block observed following pupil dilation.

The uncooperative nature of children usually makes the management of an acute attack of angle-closure glaucoma very difficult in pediatric patients [6]. Both ocular examination and treatment require sedation. In this case, IOP was not controlled by the initial medical treatment following an acute attack of pupillary block. Laser iridotomy was not easy to be applied in children. Because the visual prognosis was critical in this one-eye case, we assessed all possible surgical options, including the filtering procedure, glaucoma drainage tube, diode cycloablation, and the last resolution, clear lens extraction. The patient was encircled with a prior scleral buckle, which may have posed a technical obstacle during the surgical manipulation. Therefore, a drainage device was not considered in this case. The cycloablative procedure, though effective, may have compromised this patient’s vision. Although phacoemulsification was reported to be effective in reducing IOP in medically uncontrolled chronic angle closure glaucoma eyes [7], there has been no report of clear lens extraction in management of acute angle closure in pediatric patient, especially for such a complicated, single eye case of ROP. Finally, a filtering surgery consisting of trabeculectomy with mitomycin C treatment was decided upon after consideration of the pre-existing conjunctival fibrosis induced by the previous surgical procedure.

With the advances in neonatology, the survival rate of premature infants with low birth weight and gestational age has increased. This is associated with an increased incidence of subsequent ROP. According to a prospective study conducted at our medical center [8], the incidence of ROP and treatment-requiring ROP in all patients with ROP were 29.7 % and 37.2 %, respectively. Gestational age at birth and birth weight were the most important factors associated with treatment-requiring ROP. Regular retinal evaluation under pupil dilation is the routine procedure for most of ophthalmologists to follow the progression of ROP. An acute attack of angle-closure glaucoma is likely to occur following pupil dilation in patients with a history of ROP. Tracing back the history of this case, the patient has safely passed many sessions of pupil dilation to perform ocular examination, laser treatment, and surgical management previously. However, the episode of acute angle closure finally, and unfortunately, occurred. We suppose scleral buckling procedure may displace the lens and iris forward and aggravate the anterior chamber shallowness.

Management of acute angle-closure glaucoma in pediatric ROP is not as easy as management in adult patients. It is possible that ROP patients have some developmental structural abnormalities such as high iris convexity or posterior synechiae [9]. The anterior chamber status of ROP patients should be carefully evaluated before pupil dilation to reduce the risk of acute angle-closure glaucoma. UBM is also a good tool to evaluate the retrolental and retro-iris anatomy of ROP, although the implantation of eye cup in small patient may not be available. Alternately, a sector pupil dilation technique [10] could be tried if the patient is old enough to cooperate with the examination.

## Conclusion

Ophthalmologists should bear in mind that pupil dilation for a retina check-up could result in acute angle-closure glaucoma in ROP patients. If the anterior chamber is very shallow and the angle is very narrow, a prophylactic iridectomy should be considered in such patients.

## Consent

Written informed consent was obtained from legal guardians of the patient for publication of this Case report and any accompanying images. A copy of the written consent is available for review by the Editor of this journal.
